# Correlation Between CT-Derived Bhalla Score and Lung Ultrasound Score in Adult Patients with Cystic Fibrosis

**DOI:** 10.3390/diagnostics16111722

**Published:** 2026-06-03

**Authors:** Roxana Stoicescu, Emil Radu Iacob, Emil Robert Stoicescu, Diana Manolescu, Cristian Oancea, Alexandru Crisan, Adelina Maritescu, Alexandra Magdalena Ioana, Amalia Constantinescu, Camelia Corina Pescaru

**Affiliations:** 1 Department of Anatomy and Embriology, ‘Victor Babes’ University of Medicine and Pharmacy Timisoara, 300041 Timisoara, Romania; roxana.iacob@umft.ro (R.S.); alexandra.ioana@umft.ro (A.M.I.); 2 Research Center for Medical Communication, ‘Victor Babes’ University of Medicine and Pharmacy Timisoara, Eftimie Murgu Square No. 2, 300041 Timisoara, Romania; 3 Department of Pediatric Surgery, ‘Victor Babes’ University of Medicine and Pharmacy Timisoara, 300041 Timisoara, Romania; radueiacob@umft.ro; 4 Center for Multidisciplinary Research and Management in Pediatric Surgical Conditions, Iosif Nemoianu Street No. 2, 300011 Timisoara, Romania; 5 Department of Radiology and Medical Imaging, ‘Victor Babes’ University of Medicine and Pharmacy Timisoara, Eftimie Murgu Square No. 2, 300041 Timisoara, Romania; amalia.constantinescu@umft.ro; 6 Center for Research and Innovation in Precision Medicine of Respiratory Diseases (CRIPMRD), ‘Victor Babes’ University of Medicine and Pharmacy Timisoara, 300041 Timisoara, Romania; oancea@umft.ro (C.O.); pescaru.camelia@umft.ro (C.C.P.); 7 Department of Pulmonology, ‘Victor Babes’ University of Medicine and Pharmacy Timisoara, Eftimie Murgu Square No. 2, 300041 Timisoara, Romania; 8 Pulmonary Rehabilitation Center, Clinical Hospital of Infectious Diseases and Pulmonology “Victor Babes”, Gheorghe Adam Street 13, 300310 Timisoara, Romania; crisan@umft.ro (A.C.); adelina.maritescu@umft.ro (A.M.); 9 Research Center for the Assessment of Human Motion, Functionality and Disability (CEMFD), ‘Victor Babes’ University of Medicine and Pharmacy Timisoara, Eftimie Murgu Square No. 2, 300041 Timisoara, Romania

**Keywords:** cystic fibrosis, CTFR gene, bronchiectasis, chest computed tomography, Bhalla score, disease severity assessment

## Abstract

**Background/Objectives:** Cystic fibrosis is a chronic multisystem disease in which pulmonary involvement is the main determinant of morbidity and mortality. Chest computed tomography is the reference standard for assessing structural lung damage, but its repeated use is limited by cumulative radiation exposure. Lung ultrasound has emerged as a radiation-free alternative; however, its role in adult patients remains incompletely defined. This study aimed to evaluate the correlation between CT-derived structural lung damage and ultrasound findings, and to assess the complementary role of these imaging modalities. **Methods:** A prospective cohort study was conducted including adult patients with cystic fibrosis who underwent both chest computed tomography and lung ultrasound during the same clinical episode. Structural lung involvement was assessed using the Bhalla score, while lung aeration was evaluated using the Lung Ultrasound Score. Correlation analyses, severity stratification, regression modeling, and longitudinal comparisons were performed. **Results:** Thirteen patients contributed 24 imaging evaluations. A strong positive correlation between Bhalla score and ultrasound findings was observed in the cross-sectional analysis and remained consistent when all examinations were included. Ultrasound scores increased significantly across CT-defined severity groups, and regression analysis confirmed a significant association between the two methods. Exploratory analysis showed stronger associations for peripheral and aeration-related abnormalities, while weaker associations were observed for deeper airway changes. No significant correlation was identified in longitudinal analysis. **Conclusions:** Lung ultrasound correlates well with CT-derived structural lung damage and may serve as a complementary, radiation-free tool for disease assessment in adult cystic fibrosis. However, its limited sensitivity in detecting temporal changes highlights the continued importance of CT in selected clinical scenarios.

## 1. Introduction

Cystic fibrosis is an autosomal recessive genetic condition resulting from mutations in the CFTR gene (cystic fibrosis transmembrane conductance regulator), a protein that facilitates chloride ion transport across epithelial membranes [1,2,3]. When this channel is dysfunctional, altered ion transport results in the production of thick, viscous secretions, leading to progressive respiratory and gastrointestinal involvement [4]. This genetic disorder is one of the most common ones that might decrease the lifespan of an individual in Caucasians. It occurs in about 1 in 2500–3500 live births in European populations [5,6].

Pulmonary involvement is the primary factor influencing morbidity and mortality in cystic fibrosis [7,8,9]. In children, the condition often presents with recurrent respiratory infections, a persistent cough, and airway obstruction due to mucus plugging [10]. In adulthood, it is characterized by progressive bronchiectasis, chronic bacterial colonization, and a steady loss in pulmonary function [11,12]. As a chronic progressive disorder, cystic fibrosis places a significant burden on patients, caregivers, and healthcare systems due to the need for lifelong monitoring and recurrent therapeutic interventions [1].

Imaging is very important for diagnosing lung disease in cystic fibrosis. It may find and describe structural problems, measure the severity of the disease, and monitor of whether the condition gets worse over time [13]. Chest computed tomography remains the most effective technique to get an in-depth examination of changes in the structure of the lungs [14,15]. It gives a clear picture of bronchiectasis, mucus plugging, and parenchymal abnormalities [16]. But frequent use is limited by cumulative radiation exposure, especially in young patients who need follow-up for the rest of their lives [13].

The emergence of lung ultrasound as a radiation-free imaging modality that can be administered at the bedside and repeated as necessary is promising in this context. It provides real-time information on lung aeration and peripheral pulmonary abnormalities, making it a valuable radiation-free modality for disease assessment and follow-up [17]. However, ultrasound is intrinsically restricted in its ability to evaluate deeper lung structures, and its function in cystic fibrosis is still not fully determined [6].

The Bhalla score is a commonly used semi-quantitative CT-based scoring system that makes it possible to compare the severity of structural lung disease in a standard way [18]. The Lung Ultrasound Score (LUSS) also offers a semi-quantitative measure of lung aeration derived from ultrasound results, enabling a systematic evaluation of pulmonary involvement [19,20].

Although lung ultrasound is being used more in clinical practice, evidence supporting its ability to reflect CT-defined structural lung damage in adults with cystic fibrosis remains limited, and its role in long-term surveillance remains uncertain [6,21]. Specifically, the degree to which ultrasound findings align with established CT-based scoring systems has not been adequately investigated [6].

The objective of this study was to assess adult patients with cystic fibrosis utilizing both the Bhalla score and LUSS, to examine the association between these two methodologies in evaluating lung involvement. The study also aimed to pinpoint the benefits and drawbacks of each imaging modality and to facilitate the creation of a systematic method for their effective application in clinical follow-up.

## 2. Materials and Methods

### 2.1. Study Design and Population

A prospective cohort study was conducted at the ‘Victor Babeș’ Clinical Hospital of Infectious Diseases and Pneumophthisiology Timișoara, Romania. Adult patients with a confirmed diagnosis of cystic fibrosis were consecutively enrolled between 2023 and 2025, according to a predefined study protocol.

All eligible patients admitted during the study interval underwent standardized imaging assessments, including thoracic ultrasound and chest CT performed in parallel, with data collected in real time. Chest CT examinations were evaluated using the Bhalla score, while lung ultrasound findings were assessed using LUSS. Patient enrollment and data acquisition were performed at the time of clinical evaluation through the hospital departments involved in the management of cystic fibrosis.

After eligibility assessment, all patients meeting the inclusion criteria were included in the final analysis. The study protocol was approved by the institutional Ethics Committee, and all participants provided written informed consent (Approval no. 4989/30 May 2023).

### 2.2. Eligibility Criteria

Patients were included based on a confirmed diagnosis of cystic fibrosis and clinical indication for thoracic imaging evaluation at presentation, with imaging findings systematically recorded according to the study protocol.

Inclusion criteria

Patients were eligible for inclusion if they met all of the following criteria:

1. Age ≥ 18 years;
2. Confirmed diagnosis of cystic fibrosis;
3. Hospital admission or clinical evaluation within the study period;
4. Availability of complete clinical and imaging data;
5. Performance of both thoracic ultrasound and computed tomography according to the predefined protocol;
6. Imaging examinations performed within the same clinical episode;
7. Written informed consent obtained prior to inclusion.

Exclusion criteria

Patients were excluded if any of the following conditions were present:

1. Refusal or absence of informed consent;
2. Performance of only one imaging modality (either CT or ultrasound), without completion of both;
3. Significant time interval between imaging methods preventing reliable comparison;
4. Incomplete or non-standardized imaging protocol;
5. Poor image quality limiting interpretation;
6. Presence of other major pulmonary conditions unrelated to cystic fibrosis that could significantly confound imaging findings (e.g., advanced malignancy, extensive pulmonary fibrosis of another etiology);
7. Incomplete clinical data or loss to follow-up preventing adequate analysis.

### 2.3. Imaging Protocol and Image Analysis

Chest CT and LUSS examinations were performed on the same day for all participants to avoid potential differences related to disease exacerbations, acute viral or bacterial infections, or other temporal changes that could influence pulmonary findings.

Chest CT examinations were acquired using a 128-slice CT scanner (GE Revolution EVO, GE HealthCare, Chicago, IL, USA) and independently evaluated by two radiologists with more than 10 years of experience in thoracic imaging. Both readers were blinded to the LUSS findings.

All lung ultrasound examinations were performed using the same ultrasound system (Philips EPIQ Elite 5, Philips Healthcare, Best, The Netherlands). Depending on chest wall thickness, either a high-frequency linear transducer (used preferentially in patients with a thin chest wall) or a convex transducer (used in patients with a thicker chest wall to ensure adequate penetration) was employed. All examinations were performed and interpreted by a radiologist with more than 6 years of experience in lung ultrasound and pulmonary imaging, who was blinded to the CT images and CT-derived scores.

Lung ultrasound examinations were performed using a standardized 12-zone scanning protocol. Patients were examined in the sitting position, allowing systematic evaluation of the anterior, lateral, and posterior lung surfaces. Each hemithorax was divided into six regions (anterior superior, anterior inferior, lateral superior, lateral inferior, posterior superior, and posterior inferior), resulting in a total of 12 lung zones. The nipple line was used to separate superior from inferior regions. All image assessments were conducted independently to ensure objective scoring and minimize observer bias.

### 2.4. Variables and Definitions

The primary objective of the study was to evaluate the relationship between structural lung changes assessed by chest CT and lung aeration abnormalities detected by ultrasound in adult patients with cystic fibrosis.

The main variables analyzed were the Bhalla score and LUSS.

The Bhalla score is a validated semi-quantitative CT-based scoring system designed to assess the extent and severity of structural lung abnormalities in cystic fibrosis. It incorporates multiple parameters, including the presence and severity of bronchiectasis, peribronchial thickening, mucus plugging, sacculations or abscesses, generation of bronchial divisions involved, emphysema, and collapse or consolidation. The total score reflects the global burden of structural lung disease, with higher values indicating more severe involvement. A modified Bhalla scoring system was used throughout the study, with higher scores corresponding to greater structural lung damage.

**Figure 1:**
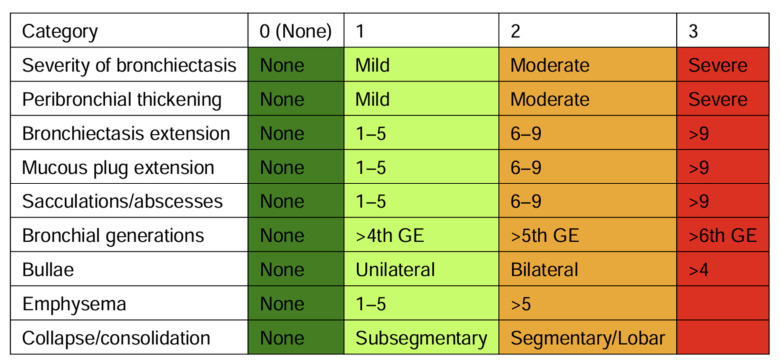
Bhalla Scoring System and Score Interpretation.

The following figure presents the Bhalla scoring system and the interpretation of each individual score component (Figure 1).

**Figure 2:**
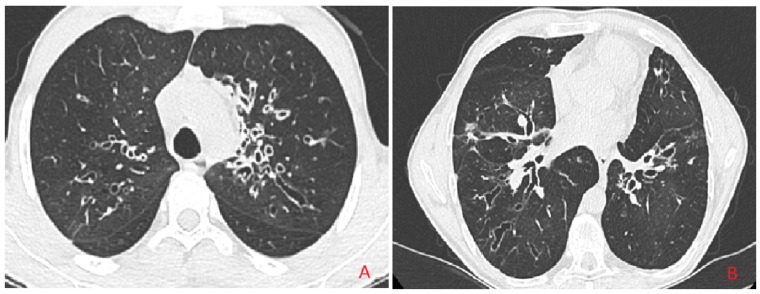
(**A**) Axial chest CT of a patient with cystic fibrosis demonstrating bilateral bronchiectatic changes, predominantly involving the left lung, with associated peribronchial wall thickening and mucus plugging. (**B**) Axial chest CT of a patient with more advanced disease, showing extensive bilateral cylindrical and saccular bronchiectasis, marked peribronchial thickening, and mucus plugging.

Below are representative axial lung-window chest CT images from patients included in the study, demonstrating the main structural pulmonary abnormalities assessed using the Bhalla scoring system (Figure 2A,B).

LUSS was used as a semi-quantitative measure of lung aeration. The score is based on the evaluation of predefined thoracic regions, taking into account ultrasound patterns such as A-lines (normal aeration), multiple B-lines (interstitial syndrome), coalescent B-lines, and subpleural or larger consolidations. Each region is assigned a score reflecting the degree of aeration loss, and the total LUSS represents the cumulative extent of pulmonary involvement, with higher values corresponding to more severe aeration impairment.

The following table (Table 1) illustrates the LUSS used in cystic fibrosis, together with representative reference images for each scoring category.

In addition to the global scores, individual CT components (including bronchiectasis, mucus plugging, consolidations, air trapping, and tree-in-bud pattern) were extracted and analyzed in relation to LUSS in exploratory analyses.

For patients with multiple imaging assessments, two analytical approaches were employed:

• Cross-sectional analysis, based on the first available evaluation per patient, to ensure statistical independence of observations.
• Longitudinal analysis, based on the comparison between the first and last available evaluations, to assess changes over time.

### 2.5. Statistical Analysis

Statistical analysis was conducted following standard methodologies commonly implemented in MedCalc^®^ Statistical Software version 23.0.9 (MedCalc Software Ltd., Ostend, Belgium), while graphical representations were generated using Python version 3.11 (pandas/SciPy) (Python Software Foundation, Wilmington, DE, USA) to ensure high-quality visualization.

Continuous variables were initially assessed for normality using the Shapiro–Wilk test. Depending on distribution, data were reported as mean ± standard deviation (SD) for normally distributed variables or median with interquartile range (IQR) for non-normally distributed variables.

The primary analysis focused on the relationship between Bhalla score and LUSS. Given the ordinal and semi-quantitative nature of both scores, Spearman’s rank correlation coefficient (ρ) was used as the main measure of association. For completeness, Pearson’s correlation coefficient (r) was also calculated when assumptions of normality were reasonably met.

To minimize bias related to repeated measurements, the primary analysis was restricted to the first available examination per patient. A secondary exploratory analysis including all available examinations was also performed.

To evaluate the clinical relevance of the relationship between CT and ultrasound findings, patients were stratified into severity groups based on Bhalla score tertiles. Differences in LUSS across these groups were assessed using the Kruskal–Wallis test, followed by post hoc comparisons where appropriate.

The association between CT-derived structural damage and ultrasound findings was further explored using linear regression analysis, with LUSS as the dependent variable and Bhalla score as the independent variable. To account for intra-patient correlation in repeated measurements, cluster-robust standard errors were applied at the patient level.

For longitudinal analysis, changes in Bhalla score and LUSS were calculated as the difference (Δ) between the first and last available evaluations for each patient. The relationship between these changes was assessed using Spearman correlation.

A two-tailed *p*-value < 0.05 was considered statistically significant.

## 3. Results

### 3.1. Study Population

A total of 13 adult patients with cystic fibrosis were included in the study, contributing 24 imaging evaluations. Among these, 8 patients (61.5%) underwent repeated assessments, enabling longitudinal analysis.

The study population consisted of 7 males (53.8%) and 6 females (46.2%). The mean age was 22.3 ± 4.9 years, with a range between 18 and 34 years, reflecting a relatively young adult cohort. The following table (Table 2) represents the baseline characteristics of the study population.

### 3.2. Distribution of Imaging Scores

Both Bhalla score and LUSS demonstrated variability across the study population, reflecting heterogeneous degrees of pulmonary involvement.

Normality testing using the Shapiro–Wilk test indicated that LUSS values were not normally distributed, whereas Bhalla scores showed a distribution closer to normality. Consequently, non-parametric statistical methods were primarily used for correlation analyses. The observed variability suggests a broad spectrum of disease severity within the study cohort, supporting the applicability of both imaging modalities across different stages of pulmonary involvement.

### 3.3. Correlation Between Bhalla Score and LUSS

#### 3.3.1. Primary Cross-Sectional Analysis

In the analysis restricted to the first available evaluation per patient (*n* = 13), a strong and statistically significant positive correlation was identified between the Bhalla score and LUSS:

• Spearman’s ρ = 0.847, *p* = 0.0003
• Pearson’s r = 0.854, *p* = 0.0002

These findings indicate that higher CT-derived structural lung damage is strongly associated with increased ultrasound-detected aeration loss.

The following table (Table 3) represents the correlation between Bhalla score and LUSS.

#### 3.3.2. Secondary Analysis (All Examinations)

When all available examinations were considered (*n* = 24), the strength of the association remained high:

• Spearman’s ρ = 0.800, *p* = 0.000003

**Figure 3:**
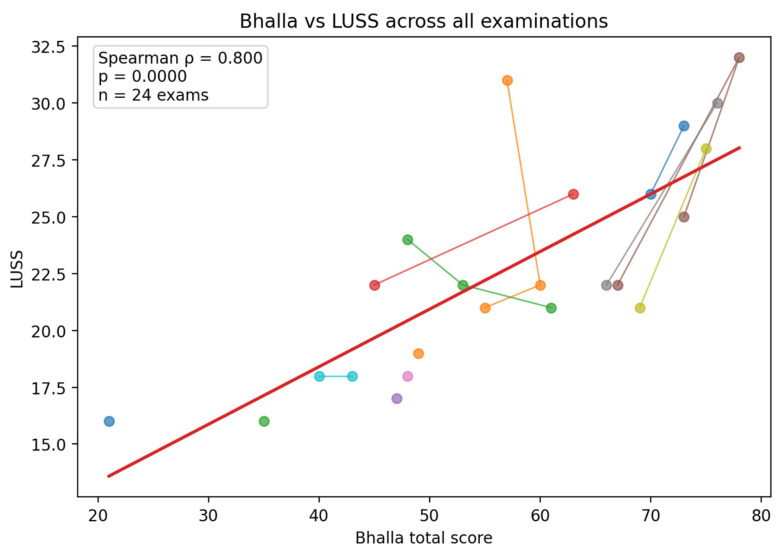
Correlation between Bhalla score and LUSS including all available examinations.

This supports the robustness of the relationship, despite the inclusion of repeated measurements. The following figure (Figure 3) represents the correlation between Bhalla score and LUSS including all available examinations.

### 3.4. Severity-Stratified Analysis

Patients were stratified into tertiles based on Bhalla score values. A statistically significant difference in LUSS across these severity groups was observed:

• Kruskal–Wallis test: *p* = 0.0298

LUSS values increased progressively with higher CT-defined disease severity, supporting the clinical relevance of ultrasound in reflecting the extent of pulmonary involvement. This trend further supports the ability of ultrasound to discriminate between different levels of structural lung disease severity.

### 3.5. Regression Analysis

Linear regression analysis demonstrated a significant association between Bhalla score and LUSS:

• Regression coefficient (β) = 0.253
• 95% confidence interval: 0.199–0.308
• *p* < 0.001

This indicates that each unit increase in Bhalla score is associated with a corresponding increase in LUSS, reinforcing the quantitative relationship between structural and functional imaging findings.

### 3.6. Exploratory Analysis of CT Subcomponents

Among the evaluated components, consolidation/atelectasis showed the strongest positive correlation with LUSS (Spearman’s ρ = 0.43, *p* = 0.036), indicating that ultrasound is particularly sensitive to structural changes associated with loss of aeration at the subpleural level. Air trapping demonstrated a moderate but non-significant correlation (ρ = 0.33, *p* = 0.113), while bronchial consolidation showed a weaker association (ρ = 0.29, *p* = 0.163).

Tree-in-bud pattern (ρ = 0.23, *p* = 0.277) and saccular bronchiectasis (ρ = 0.21, *p* = 0.334) exhibited weak correlations with LUSS, suggesting limited ultrasound sensitivity for small airway and deeper parenchymal abnormalities. Similarly, bronchial wall thickening showed minimal or inconsistent associations, with severe thickening demonstrating a weak positive correlation (ρ = 0.10, *p* = 0.632), while mild thickening showed a weak negative correlation (ρ = −0.28, *p* = 0.185), both without statistical significance.

Overall, these findings indicate that LUSS is more strongly associated with CT features that produce peripheral or subpleural alterations and measurable aeration loss, while its ability to detect deeper or early structural changes remains limited.

**Figure 4:**
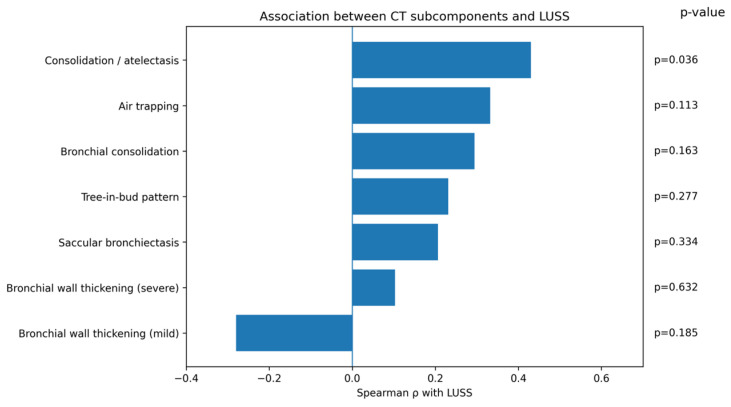
Correlation between CT structural components and LUSS. Horizontal bars represent Spearman correlation coefficients (ρ) for each CT feature, while corresponding *p*-values are displayed separately. Stronger associations were observed for bronchiectasis, mucus plugging, and consolidations, whereas weaker correlations were noted for air trapping and tree-in-bud pattern.

The following figure shows the association between CT subcomponents and LUSS (Figure 4).

### 3.7. Longitudinal Analysis

In the subgroup of patients with repeated evaluations (*n* = 8), the relationship between changes in Bhalla score and changes in LUSS was not statistically significant:

• Spearman’s ρ = 0.370, *p* = 0.367

Similarly, paired comparisons between the first and last evaluations did not demonstrate statistically significant differences for either Bhalla score or LUSS. The limited magnitude of changes observed between evaluations may partially explain the lack of a statistically significant association.

**Figure 5:**
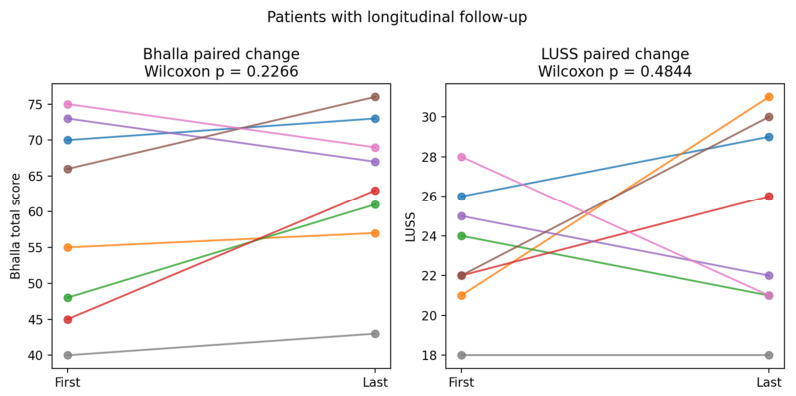
Paired comparison between first and last evaluations for Bhalla score and LUSS.

The following figure shows the paired comparison between the first and last evaluations for Bhalla score and LUSS (Figure 5).

## 4. Discussion

The present study demonstrated a strong correlation between the Bhalla score and LUSS in adult patients with cystic fibrosis, suggesting that lung ultrasound reflects CT-defined disease burden. While CT remains the reference standard for structural lung evaluation, these findings support the complementary role of LUSS as a radiation-free imaging tool for disease assessment. A major strength of this study is that, to the best of our knowledge, it is the first to assess the relationship between chest CT and lung ultrasound in adults with cystic fibrosis using a standardized imaging protocol. Although similar findings have been reported in pediatric cohorts, data in adults remain limited [22,23]. By addressing this gap, our findings extend the applicability of lung ultrasound to adults with cystic fibrosis and demonstrate that LUSS closely reflects CT-defined disease burden, supporting its complementary role in imaging assessment. Nonetheless, the longitudinal analysis did not reveal a significant correlation between changes in the Bhalla score and LUSS, suggesting limited sensitivity of ultrasound to subtle temporal disease changes. This finding may be explained by the fact that CT and LUSS evaluate different aspects of pulmonary pathology, together with the limited magnitude of imaging changes observed over time and the small sample size. Therefore, while the present findings support the value of LUSS for cross-sectional disease assessment, they do not provide sufficient evidence to establish its reliability for monitoring longitudinal disease progression.

Analysis of individual CT components showed stronger correlations for peripheral and subpleural abnormalities and weaker correlations for deeper airway changes. These findings support the role of LUSS in detecting peripheral lung disease while confirming the complementary value of CT for comprehensive structural evaluation. These observations align with findings in pediatric populations, where lung ultrasound has demonstrated a strong correlation with CT-based assessments and excels in identifying peripheral lesions, such as consolidations and atelectasis, while being less effective in evaluating deeper abnormalities [22]. The consistency of these findings across different age groups supports the reliability of ultrasound as an additional imaging technique for cystic fibrosis.

Additionally, prior research assessing CT-based scoring systems has shown that structural lung damage, as measured by modified Bhalla scores, correlates with clinical and functional parameters, including pulmonary function tests [18]. These findings support the clinical relevance of CT-derived structural assessment. The correlation observed between CT and LUSS findings further highlights the potential role of ultrasound as a complementary monitoring tool in adult cystic fibrosis. Furthermore, research evaluating the diagnostic efficacy of lung ultrasound in cystic fibrosis has indicated a high accuracy in identifying peripheral abnormalities, including consolidations and atelectasis, while exhibiting inconsistent performance regarding interstitial or deeper parenchymal alterations [24,25]. These findings support the complementary roles of ultrasound and CT in the assessment of cystic fibrosis lung disease.

Overall, these findings support a complementary imaging approach in cystic fibrosis, with CT providing comprehensive structural assessment and lung ultrasound offering a radiation-free option for bedside evaluation and follow-up. Beyond lung ultrasound, chest MRI is increasingly emerging as a valuable radiation-free imaging modality for the assessment of cystic fibrosis lung disease. While CT remains the reference standard for detailed structural evaluation, MRI offers the additional advantage of repeated examinations without ionizing radiation and the potential to provide both structural and functional information. However, limitations related to availability, examination time, cost, and protocol variability currently restrict its widespread implementation. Rather than competing modalities, CT, MRI, and lung ultrasound should be viewed as complementary tools, each providing unique information that may contribute to a more comprehensive evaluation of disease status. Future efforts should focus on the development of standardized multimodal imaging protocols that define the optimal role and timing of each modality according to disease severity, clinical status, and follow-up requirements, thereby optimizing patient monitoring while minimizing cumulative radiation exposure [26].

This study is limited by its small sample size, single-center design, and variability in follow-up intervals, which may affect the generalizability and longitudinal interpretation of the findings. However, to our knowledge, this is the first study comparing chest CT and lung ultrasound exclusively in adult patients with cystic fibrosis using a standardized imaging protocol. Finally, both CT and LUSS scores are semi-quantitative and therefore subject to some degree of observer dependence. Future studies should validate these findings in larger multicenter cohorts and integrate imaging results with pulmonary function, clinical outcomes, and exacerbation data. Advanced approaches such as radiomics and AI may improve the detection of subtle disease changes and support prognostic modeling. Integrated multimodal strategies combining CT, lung ultrasound, and chest MRI may further optimize personalized monitoring while minimizing radiation exposure.

## 5. Conclusions

Lung ultrasound demonstrates a strong association with CT-derived structural lung damage, as assessed by the Bhalla score, in adult patients with cystic fibrosis, supporting its role as a reliable indicator of disease severity in a cross-sectional setting. Its ability to reflect peripheral and aeration-related changes makes it a valuable, radiation-free tool for disease assessment and bedside evaluation. However, its limited sensitivity in detecting subtle temporal changes and deeper airway abnormalities underscores the continued importance of CT for comprehensive structural assessment. Further longitudinal studies are required to determine its role in monitoring disease progression over time. Overall, a complementary imaging approach integrating both CT and lung ultrasound may optimize patient evaluation and follow-up, balancing diagnostic accuracy with the need to minimize radiation exposure.

## Figures and Tables

**Table 1:** LUSS in Cystic Fibrosis: Reference Images and Scoring Criteria.

LUSS Artefact	Lung CF Score	LUSS Finding—Corresponding Image
Presence of A lines—normal aspect Distinctive B lines <3/intercostal (ic) space	0	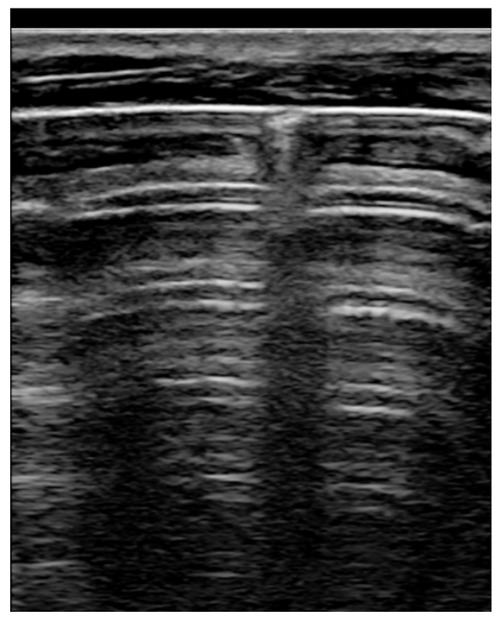
Distinctive B lines >3/ic space or 1 coalescent B line	1	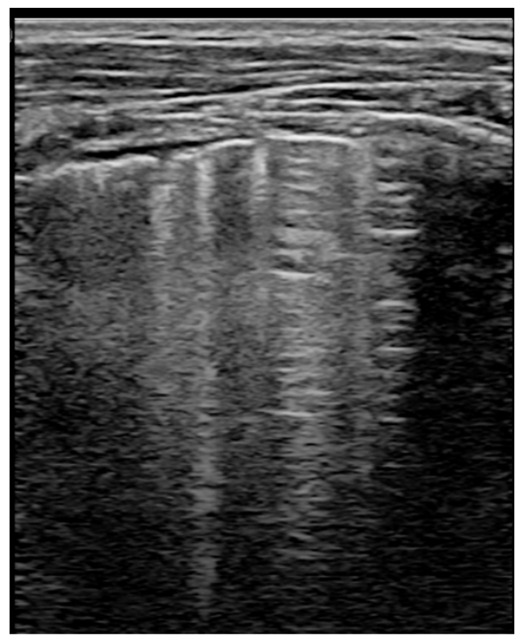
Coalescent B lines >2/ic space	2	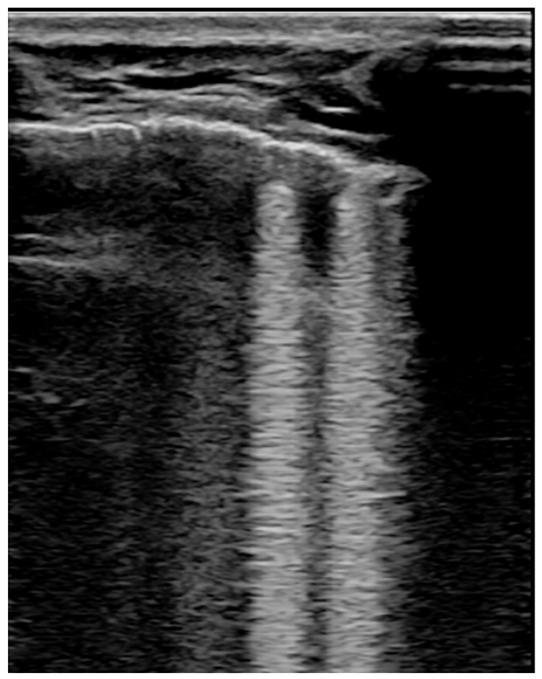
Consolidation <1 cm	3	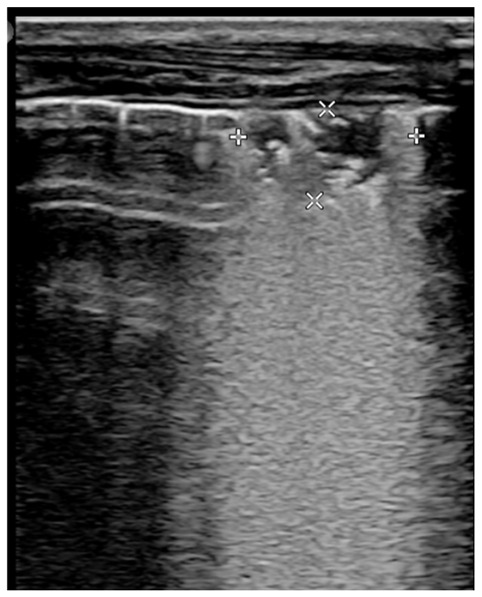
Consolidation >1 cm, with bronchogram	4	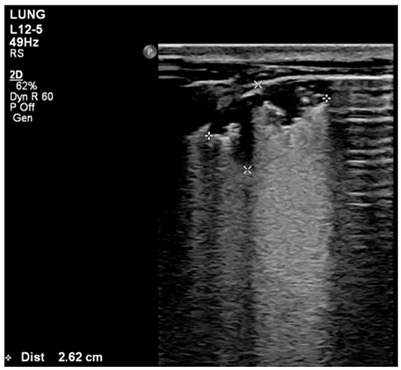
Atelectasis/consolidation without bronchogram, >1 cm	5	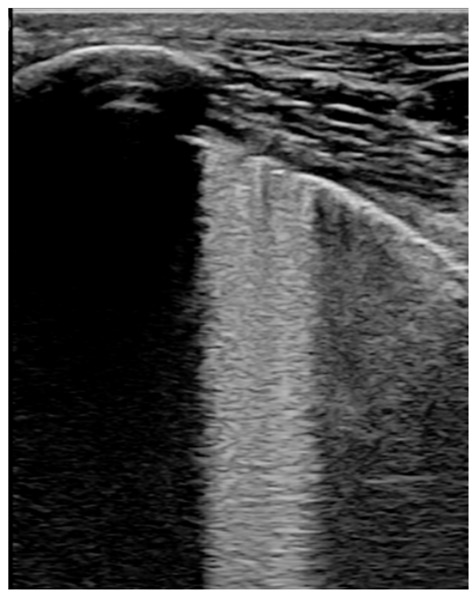

**Table 2:** Baseline characteristics of the study population.

Variable	Value
Number of patients	13
Sex, *n* (%)	
Male	7 (53.8%)
Female	6 (46.2%)
Age, years (mean ± SD)	22.3 ± 4.9
Age range, years	18–34
Number of imaging evaluations	24
Patients with follow-up, *n* (%)	8 (61.5%)

**Table 3:** Correlation between Bhalla score and LUSS.

Analysis	Correlation Coefficient	*p*-Value
Baseline (Spearman)	0.847	0.0003
Baseline (Pearson)	0.854	0.0002
All examinations (Spearman)	0.800	<0.001

## Data Availability

The original contributions presented in this study are included in the article. Further inquiries can be directed to the corresponding authors.

## Abbreviations

The following abbreviations are used in this manuscript:

CFTR	cystic fibrosis transmembrane conductance regulator
CT	computed tomography
ic	intercostal
LUSS	Lung Ultrasound Score
SD	standard deviation
